# Electrically Enhanced Sensitivity (EES) of Ion-Selective Membrane Electrodes and Membrane-Based Ion Sensors

**DOI:** 10.3390/membranes12080763

**Published:** 2022-08-03

**Authors:** Jan Migdalski, Andrzej Lewenstam

**Affiliations:** Faculty of Materials Science and Ceramics, AGH-University of Science and Technology, Al. Mickiewicza 30, 30-059 Cracow, Poland; migdal@agh.edu.pl

**Keywords:** open-circuit, summation, membranes, ion-sensor, solid contact, multielectrode platform, sensitivity, slope, determination error, S/N ratio

## Abstract

The use of external electronic enforcement in ion-sensor measurements is described. The objective is to improve the open-circuit (potentiometric) sensitivity of ion sensors. The sensitivity determines the precision of analyte determination and has been of interest since the beginning of ion-sensor technology. Owing to the theoretical interpretation founded by W.E. Nernst, the sensitivity is characterized by the slope and numerically predicted. It is empirically determined and validated during calibration by measuring an electromotive force between the ion sensor and the reference electrode. In practice, this measurement is made with commercial potentiometers that function as unaltered “black boxes”. This report demonstrates that by gaining access to a meter’s electrical systems and allowing for versatile signal summations, the empirical slope can be increased favorably. To prove the validity of the approach presented, flow-through ion-sensor blocks used in routine measurements of blood electrolytes (Na^+^, K^+^, Li^+^, Cl^−^) and multielectrode probes with flat surfaces, similar to those applied previously for monitoring transmembrane fluxes of Na^+^, K^+^, Cl^−^ through living biological cells, are used. Several options to serve real-life electroanalytical challenges, including linear calibration for sensors with high-resistance membranes, responses with non-Nernstian slopes, non-linear calibration, and discrimination of nonfunctional sensors, are shown.

## 1. Introduction 

Ion-selective membrane electrodes (ISEs) and ion-sensitive sensors (ISs) (i.e., the ISEs with solid-state contact) [1] in potentiometric (zero-current) mode are commonly applied owing to several advantages. Among them are wide concentration ranges, good ion discrimination, possible direct measurement, fast response, simplicity of construction, easy maintenance, and low price. Moreover, the easy-to-measure potentiometric signal does not depend on the geometric size of the sensing membrane surface, which remains in contrast to non-zero-current electroanalytical sensing. Finding the best analytical performance of electrochemical ion-selective electrodes has been the driving force behind research the last several decades. Dedicated improvements in membrane chemistry, internal contact design, sensor architectures, and analytical measurement procedures have been presented in thousands of reports [2,3].

The parameters describing the response of the ion-sensitive probes are now supported both by advanced theories (such as modelling in space and time domains [4]) and by the employment of inventive fabrication technologies (such as 3D sensor printing). Owing to these advancements, non-equilibrium potentiometry and computer-aided 3D sensor casting are feasible, which greatly contributes to a variety of ISE applications [5], including routine and large-scale applications in the clinical analysis of blood electrolytes [6].

A milestone in the design of ISEs was the introduction of a solid contact (SC) instead of an internal liquid solution in 1992 (see [2,7]), which resulted in the design of all-solid-state ion-sensors (SC-ISs). Owing to this invention, new designs allowing sensors to be miniaturized, placed in any position, and making them maintenance-free and easy-storing, were offered. SC-ISs promoted the modern technology of multielectrode flow-through blocks and flat sensing multi-platforms, promoting their applicability in electrolyte monitoring and screening, for instance, by wearable belts [7,8,9,10,11,12,13,14,15].

Both ISEs and SC-ISs have the same ion-sensitive membrane (ISM), and both co-operate with a potentiometer. This electrical co-partner of the potentiometric measurement is, as a rule, a “black box” for an average user of sensors. Applied as delivered, it allows for empirical evaluation of ion-sensor sensitivity characterized by the calibration slope, but as a rule, it is not used or involved in the interventions aimed to increase the slope.

The reason is scientifically significant. In the open circuit, the ion-sensitivity of a single ion-sensor in an equilibrium mode is characterized theoretically by a slope factor, “s”, where s = RT/zF (R is the gas constant, T is absolute temperature, F is the Faraday constant, and z is a charge of an ion), and s = 25.69/z mV at 298.15 K. The theoretical interpretation of the slope originates from basic thermodynamics and is provided by the Nernstian function E vs s ln (a_i_) where E is electric potential and a_i_ ion activity. The compliant slope given by the Nernst equation with decimal logarithmic function, is called the Nernstian slope, where S = 2.30 s = 59.16/z mV at room temperature (298.15 K) [1,5]. The theory directly predicts slope-dependent determination error in potentiometric measurement of ions by a single ion sensor [9,16]. The theoretical determination error (DE), in direct ion potentiometry, is DE = ∆E/s = 2.30 ∆E/S, where ∆E is a potential readout resolution (uncertainty) in mV. Thus, for ∆E = 1 mV, the DE (%) is ~4% for monovalent ions and ~8% for divalent ions.

In empirical practice, the apparent slope differs from the theoretical one. It is typically sub-Nernstian owing to membrane-related processes, sample-related interferences, and composition [9,16]. Nevertheless, in principle the same interpretation as the theoretical given above, where DE is inversely proportional to the slope, holds. Thus, enhancing sensitivity offers a very advantageous prospect that can provide direct benefits, because it particularly contributes to increased precision. The experimental challenge of sensitivity is identified but routinely addressed outside the meter and determined by adjusting the electrochemical properties of the sensor and sample.

Addressing sensitivity via the electrical properties of the measurement system is performed very rarely. This has been achieved tentatively by Stepak [17], who applied several identical galvanic cells interconnected in series by electrochemical salt bridges, initially called Cells Connected in Series, (CCS). The electrical potential difference of the CCS is the sum of the individual cell potentials, which means that the slope increase is directly proportional to the number of individual cells in the series [17]. The same idea was recently adopted by Zdrahek and Bakker [18]. Each cell contained both an ion-selective membrane electrode and reference electrode (REF) immersed in separate solution samples with identical composition. In these reports, the potential difference in the CCS was measured by retrieving the readouts from commercial pH meters and potentiometers, left intact as they are. The practical usability of these systems is obviously limited, as shown by Parczewski and Stepak [19]. First, a large solution volume is needed for a separate distribution in each cell during calibration and measurement. Moreover, every cell in the series is presumed to work correctly and identically to allow summing the Nernstian responses {17,18]. Secondly, the resultant resistance of the cell’s system is an algebraic sum of individual cell resistances. Thus, a more significant noise level of the signal can be expected, unfavorably suppressing the signal-to-noise (S/N) ratio. The theory of error propagation presented [19] shows that the optimal application of the CCS should be expected with four cells. Indeed, using four copper (II) ISEs with a solid membrane, the method provides better results than a single cell with Cu-ISE electrode in low concentrations of the Cu^2+^ ion, corresponding to non-linear parts of the calibration curve [20]. The CCS method is, in practice, more demanding than conventional single-cell potentiometry (SCP), which sets the basis for our motivation to seek a more attractive approach.

Our proposal goes beyond the frame characterized above. Instead of summing the electric potentials (i.e., electromotive forces) of the interconnected galvanic cells, each separately filled with the same electrolyte solution, we postulate to enhance sensitivity electronically by summing the electrical potentials of the multielectrode system using adopted external electronics (EES). The potential of each indicator electrode in the serial flow-through electrode block or a flat multielectrode platform is measured in the same solution sample against the common reference electrode and undergoes a transformation by a repeater (impedance transformer). The signals from the repeaters are summed up in order to multiply the sensitivity of the ion-sensor system. They are also used for assessing the correctness of the sensors’ response. Consequently, the suitability of each ion-sensor for further operation, including the summation of signals, is obtained. Any ion-sensor meeting the criteria of electrochemical performance is used to get a final outcome, while those sensors that do not meet the criteria are ignored. After summing up the signals of individual electrodes, the total voltage is the basis for analytical application, including calibration patterns and referring the sample results to them similarly as in conventional potentiometry.

This EES strategy translates into important practical advantages that are documented in this report. To illustrate the benefits of the EES, we deliberately selected the two experimental options. In the first one, the block containing a commercial flow-through indicator and reference electrodes, applied in high throughput clinical analyzers, is used [6]. We show the EES strategy output for the membranes with high resistance (Na glass) and for the membranes with insufficient selectivity for the main ion (Na interference on Li electrode). The other system studied is the home-made, flat-surface multielectrode probe, in which a solid contact reference electrode surrounded by ion-sensors (K, Na, Cl-ISs) work in a small volume sample. This system has been used before (see [2]) for service-free tracing of transmembrane ion fluxes, monitored for hours at the surface of leaving biological cells, or for electrolyte measurements in a single-drop sample [21]. In all the above cases, the sensitivity increases, and discrimination of the malfunctional sensors offered by the EES may support the reliability of the measurement.

## 2. Materials and Methods

### 2.1. Apparatus

**Figure A1:**
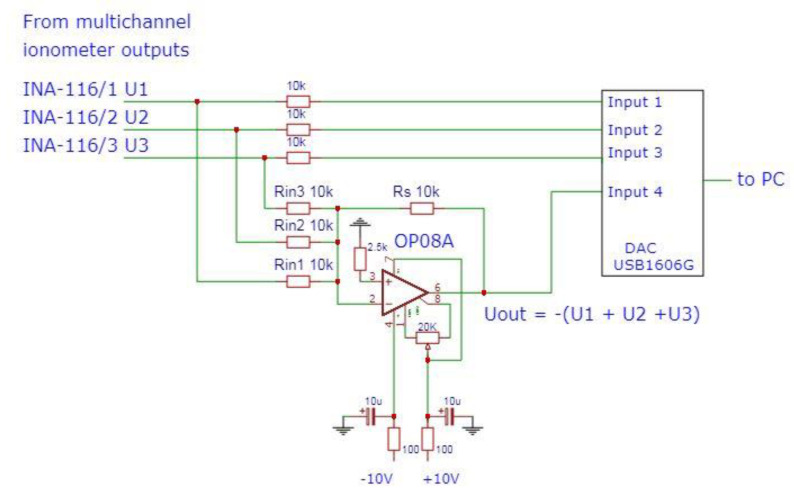
Scheme of the summing amplifier built with the Ultralow Offset Voltage Operational Amplifier OP08A (Analog Device) as well as its cooperation with a multichannel ion-meter and data acquisition card (DAC). The scheme was made with the use of free EasyEDA software.

The home-made measuring setup containing a 16-channel ion-meter with the input bias current of <3 fA and input resistance of >10^15^ Ω, cooperating with the summing amplifiers and a commercial data acquisition card (DAC) (see Appendix A, Figure A1) was used to improve the ISs sensitivity in all EES measurements. The setup allows for simultaneous tracing of up to 16 ISEs signal changes, measured versus the same reference electrode. The mean potential values presented in Table 1, Table 2, Table 3, Table 4 and Table A1 were calculated using the last 20 potential readings taken at 5 s intervals. The setup signal resolution was 0.015 mV and for this reason 0.02 mV was assumed as the minimal SD value. All measured signals were visualized and archived by a data acquisition card. Simultaneously, the signals of the selected ISEs can be summed up by inverting summing amplifiers in order to multiply the sensitivity. The inverted summed signal (i.e., multiplied by −1) was also visualized by DAC, simultaneously with the signals to be summed up. In his way, by activating an electronic control for each electrode response, any ISE malfunctions can be immediately identified, and such electrode(s) can be eliminated from summing. In contrast to the previously described methods, our setup allows for summing the ISE signals measured versus the same (single) reference electrode, with all electrodes immersed in the same solution. This way, the solution volume can be extremely reduced, e.g., up to a single drop. Because all ISE signals undergo initial transformation by a repeater (impedance transformer), the signals of the highly resistive ISEs, e.g., with a glass membrane (with membrane resistance approx. 0.5 to 1 GΩ), also can be summed up.

### 2.2. Chemicals

#### 2.2.1. ISE Membranes

Potassium ionophore I (valinomycin), tridodecylmethyammonium chloride (TDMCl), potassium tetrakis(4-chlorophenyl) borate, bis(2-ethylhexyl) sebacate (DOS), and poly(vinyl chloride) high molecular weight (PVC) were purchased from Fluka (Buchs, Switzerland). Silver nanowires of diameter 175 nm × 20–50 nm as a 0.5% suspension in isopropanol obtained from Sigma-Aldrich (Steinheim, Germany) were used as received. Tetrahydrofuran (THF) purchased from Sigma-Aldrich was double-distilled before use.

To prepare the reference cocktail [22], silver chloride and potassium chloride were dried, mixed in molar ratio 1:5, and thoroughly ground. Additionally, 2.06 g bis (2-ethylhexyl)-sebacate (DOS) and 1.24 g poly(vinyl chloride) (PVC) were dissolved in 25 mL of freshly distilled THF. Next, 1.5 mL of a PVC/DOS solution and 0.1 mL silver nanowires in isopropanol were added to 0.224 g of a AgCl/KCl mixture.

To prepare the potassium cocktail, the valinomycin (1% *w*/*w*), potassium tetrakis (4-chlorophenyl) borate (0.5% *w*/*w*), bis (2-ethylhexyl) sebacate (DOS) (65.5% *w*/*w*), and high molecular weight poly(vinylchloride) PVC (33% *w*/*w*) were dissolved in freshly distilled tetrahydrofuran (THF).

To prepare the chloride cocktail, tridodecylmethyammonium chloride (TDMACl, 20% *w*/*w*) was dissolved together with PVC in THF.

#### 2.2.2. Other Reagents

All other compounds used (LiCl, NaCl, KCl, KNO_3_, AgCl AgNO_3_) were obtained as p.a. from POCh, Poland and Merck, Germany and were used as received.

Water re-distilled from quartz was used to prepare the solutions. All solutions with concentrations lower than 0.01 mol dm^3^ were prepared just before use.

### 2.3. Electrodes

#### 2.3.1. Flow-Through Electrodes

**Figure 1:**
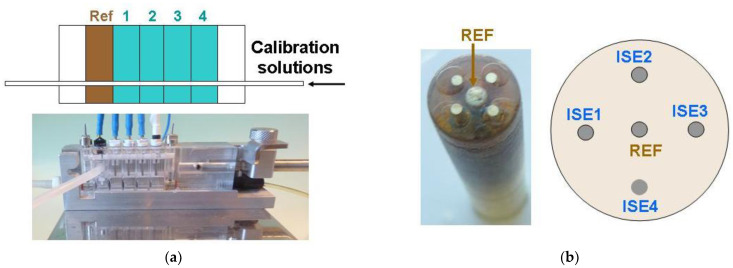
(**a**) Flow through set containing four ISE electrodes with plastic membranes and a REF electrode, all from KONE. (**b**) Multielectrode platform with an external diameter of 10 mm containing five Au/Ag disc substrates (0.5 mm diameter). The central electrode (indicated by an arrow) is a solid-contact reference electrode. It is surrounded by four SC ISs.

The flow-through commercial ISEs and REF electrodes were obtained from KONE Instruments (now Thermo Fisher Scientific, Vantaa, Finland). Lithium electrodes with a plastic membrane are referred to as Li1-Li4; sodium electrodes with a capillary glass membrane are marked as Na1, Na2, and Na3; sodium electrodes with plastic membranes are marked as Na-ISE, and the reference electrode as KONE-REF. Figure 1a shows the block of the flow-through-type electrodes. The sample volume used with the flow-through electrode blocks is 40 μL. Three glass ISEs were used in the block due to the larger own-volume of the glass capillary membrane.

#### 2.3.2. Multielectrode Platforms

A platform (probe) containing the ion-sensitive sensors and the reference electrode was prepared using an epoxy body with an external diameter of 10 mm, containing five gold substrate disc electrodes with a diameter of 0.5 mm (see Figure 1b). Prior to membrane cocktail casting, the gold substrate surfaces were electrochemically covered with silver. Then, on these Au/Ag substrates, solid-contact ISs and SC REF electrodes were prepared. The sample volume used for the measurements with the probe was 30 μL.

#### 2.3.3. Procedures

The Ag/AgCl electrode REF 201 and REF 251 (Metrohm) were used, usually to confirm the proper functioning of the KONE REF or the solid-contact REF. Mean activity coefficients were calculated using the Debye–Huckel equation and used for numerical conversion of concentrations to the corresponding activity in Table 1, Table 2, Table 3, Table 4 and Table A1, and the related calibration graphs.

## 3. Results and Discussion

According to Nernst theory, the determination error with potentiometric ion sensors is inversely proportional to the slope whose magnitude depends on the ion charge. In general practice, a truism says that the bigger the sensor slope, the better the precision is. There are several apparent situations in which an increase in the slope, as proposed herein, is highly relevant and beneficial. Increasing the slope of the sensors with highly resistive membranes, increasing sensitivity in the non-linear part of calibration caused by the influence of interfering ions (similar effect of detection limit is not shown here), and discarding malfunctioning sensors are of importance for both ion-sensor blocks and multielectrode flat systems, which are documented below.

### 3.1. Slope Amplification by Summing Signals in Flow through Blocks or Multielectrode Platforms with n-Sensors for the Same Ion

#### 3.1.1. Amplification of the Slope with Highly Resistive Sodium Glass Membrane Electrodes Set in a Flow-Through Block

**Figure 2:**
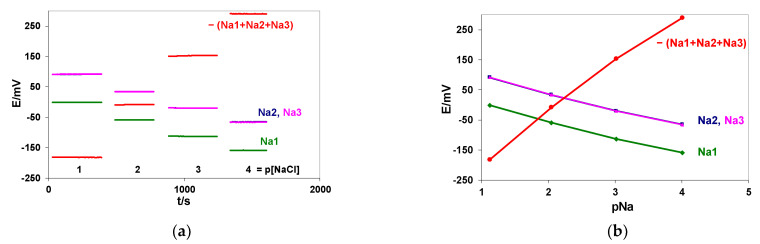
Potential changes of three highly resistive sodium electrodes with glass membranes and their summed signals (**a**). Calibration curves of three highly resistive sodium electrodes with glass membranes and of their summed signals (**b**). Measurements were performed in 0.1–0.0001 M NaCl solutions.

The CCS method proposed is limited by the internal resistance of the indicator ion-sensors. In our setup, the potential of each ISE was measured versus the same reference electrode, and all electrodes were in contact with one portion of the same solution (Figure 1a). The performance of the block containing three flow-through, highly resistive sodium electrodes with glass membranes (Na1, Na2, Na3) was measured to check the EES method applicability. Calibration was performed in NaCl solutions in the concentration range 0.1–0.0001 M. The signals of the Na1, Na2, and Na3 electrodes were measured against an external silver chloride electrode, REF 201 (Metrohm), and summed. The recorded signals and resulting calibration curves are shown in Figure 2a,b.

The detailed calibration results, calculated correlation coefficients (R), and mean signal-to-noise ratio (S/N) values are collected in Table 1. The slopes were determined for the linear parts of the calibration curves. The correlation coefficients (R) and mean values of the signal-to-noise ratios were determined for the linear part of the calibration curves. The S/N ratios were calculated according to the pattern used before in [18]. For each concentration belonging to the linear part of the calibration curve, the slope value was divided by the uncertainty of the potential readout (i.e., the SD value obtained for this concentration), and then the obtained values were summed up and averaged.

The results presented in Table 1 show that the proposed method of a slope increase by summing the signals of three commercial, highly resistive Na-ISEs with glass membranes brings about 3-times bigger slope values (compared with the signals recorded for the electrodes to be summed), resulting in a greatly improved S/N ratios. Small differences observed between the electronically summed signal and absolute values of the manually summed signals of the Na1, Na2, and Na3 electrodes (below 1mV and similar for each NaCl concentration) can be caused by a small offset voltage of the summed amplifier. The experiment shows that high resistance of the membranes can be submitted to the EES methodology, preserving the main benefit of a multiplied slope. Moreover, the method is valid even if the formal potential of the ion sensors differs, which was the case for Na1 vs. Na2 and Na3.

**Table 1:** Mean potential values (E) and their standard deviation (SD), the slope values determined for the linear parts of the calibration curves and the correlation coefficients (R) and signal-to-noise ratio (S/N) ratios are presented. The measurements were performed with the setup shown in Figure 1a, containing three flow-through, sodium-sensitive electrodes.

pNa	E/mV ± SD			
	Na1 Glass	Na2 Glass	Na3 Glass	−(Na1 + Na2 + Na3) Glass
1.12	−1.25 ± 0.03	91.52 ± 0.03	91.84 ± 0.05	−182.16 ± 0.02
2.05	−59.41 ± 0.04	33.68 ± 0.02	33.86 ± 0.03	−8.12 ± 0.04
3.02	−113.34 ± 0.06	−20.21 ± 0.06	−20.32 ± 0.05	153.78 ± 0.09
4.01	−159.04 ± 0.15	−64.99 ± 0.19	−66.39 ± 0.16	290.41 ± 0.24
**Slope mV/pNa** **pNa 1–3**	−59.18	−59.00	−59.22	177.38
**R pNa 1–3**	−0.99937	−0.99942	−0.99945	0.99941
**Mean S/N pNa 1–3**	1480	1967	1448	5092
**Potential changes between pNa 3–4**	−45.70	−44.7	−46.07	136.63

#### 3.1.2. Amplification of the Slope in the Case of Non-Linear Calibration: The Interference of Sodium Ions on Flow-Through Lithium ISEs

Lithium electrodes are routinely used in clinical blood measurements [6]. The insufficient selectivity for sodium is a challenge manifested by the sodium-dependent non-linearity of the calibration curves for the main Li^+^ ion when interpreted by the Nernst equation. This effect is formally corrected by the Nikolsky–Eisenman equation [5], which takes formally into account the interference of sodium ions and retains the Nernstian interpretation of the slope.

**Figure 3:**
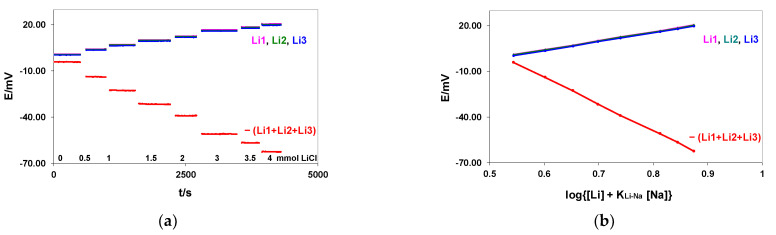
Responses of the lithium electrodes Li1, Li2, and Li3 and their summed signals recorded during LiCl addition to 140 mmol NaCl (**a**). The calibration curves of the lithium electrodes Li1, Li2, and Li3 and their summed signals (**b**). Potential changes were measured versus the flow-through KONE REF (electrode block used as shown in Figure 1a).

To show the applicability of our approach in such a case, the blocks formed by three flow-through commercial lithium electrodes (Li1, Li2, and Li3) and the KONE-REF electrode, as shown in Figure 1a, were used to test the amplification schemes. The measurements were performed in solutions containing 140 mmol/L NaCl, and different concentrations of LiCl ranged from 0 to 4 mmol/L. The signals of the Li-ISEs measured versus the KONE-REF were summed. The measurement methodology is presented in Figure 3a. The simultaneous potential difference between the external silver chloride electrode (REF-201, Metrohm) and the KONE-REF was measured to prove the proper functioning of the KONE-REF (see Table 2). The experimentally determined selectivity coefficient, K_Li,Na_, was equal to 0.0250, and therefore the calibration curves were plotted with the coordinates of the Nikolsky–Eisenman equation: E vs. log {[Li] + K_Li-Na_[Na]} = E vs. log {[Li] + 0.0250 × 140} = E vs. log [Li] + 3.5}. The calibration curves for the individual Li-ISEs and the signal sums are shown in Figure 2b.

The last Li4 electrode from the block was connected simultaneously to three other ion-meter channels with different gains equal to 1.0, 2.0, and 3.2 (to show the additional possibility of the EES method, see Appendix A.2).

Detailed calibration results, the calculated correlation coefficients (R), and the mean S/N ratio values are collected in Table 2. The simultaneously measured potential difference between the external silver chloride electrode (REF-201, Metrohm) and the KONE-REF proved the proper functioning of the KONE-REF, as illustrated in the last column in Table 2.

The results presented in Table 2 show again that the proposed method of slope increasing through summing the potentials of three individual Li-ISEs produces about a 3-times bigger signal with a 3-times bigger potential resolution (compared with the signals recorded for electrodes to be summed). The differences (about 1.9 mV, similar for each LiCl concentration) observed between the absolute values of the electronically summed signal and manually summed signals of the Li1, Li2, and Li3 electrodes can be caused by a small offset voltage of the summed amplifier. In the procedure of clinical measurement of lithium, the interference of sodium is numerically corrected by the product K_Li,Na_ [Na^+^] with independently measured sodium. Increasing the slope in the Nikolsky–Eisenman equation allows for more precise control of K_Li,Na_ in the calibration, which is necessary to get reliable lithium results.

**Table 2:** Mean potential values (E) with their standard deviation (SD), slope values, calculated correlation coefficients (R), and the mean signal-to-noise ratios (S/N) are presented. The last column shows the recorded potential difference between the external silver chloride electrode (REF-201, Metrohm) and KONE-REF (the electrode block shown in Figure 1a was used).

LiCl (mmol/L)	E/mV ± SD				
	Li1	Li2	Li3	−(Li1 + Li2 + Li3)	REF Metrohm vs. KONE REF Difference
0.0	1.12 ± 0.02	0.91 ± 0.02	0.33 ± 0.02	−4.20 ± 0.04	5.58 ± 0.02
0.5	4.36 ± 0.03	4.11 ± 0.02	3.58 ± 0.03	−13.91 ± 0.06	5.45 ± 0.02
1.0	7.31 ± 0.02	7.05 ± 0.02	6.48 ± 0.04	−22.72 ± 0.07	5.41 ± 0.02
1.5	10.30 ± 0.03	10.06 ± 0.04	9.43 ± 0.05	−31.64 ± 0.09	5.37 ± 0.02
2.0	12.80 ± 0.07	12.48 ± 0.06	11.87 ± 0.06	−39.04 ± 0.09	5.28 ± 0.04
3.0	16.77 ± 0.02	16.42 ± 0.02	15.89 ± 0.03	−50.99 ± 0.05	5.23 ± 0.02
3.5	18.70 ± 0.03	18.35 ± 0.02	17.78 ± 0.03	−56.68 ± 0.09	5.58 ± 0.02
4.0	20.66 ± 0.02	20.30 ± 0.02	19.71 ± 0.02	−62.54 ± 0.06	5.19 ± 0.02
**Slope ** **mV/Δlog [Li + 3.5]**	58.99	58.52	58.50	−176.09	
**R**	0.9944	0.9943	0.9945	−0.9944	
**Mean S/N**	2316	2499	1913	2772	

#### 3.1.3. Discriminating Malfunctioning Ion Sensors in Systems of the Flat Multielectrode ISs and Solid Contact Reference Electrode

Multielectrode platforms rapidly attracted interest in sensor technology because of the realistic prospect of applications in personal, non-invasive monitoring of electrolytes in peripheral bioliquids, e.g., extracellular liquids [2], sweat [23,24,25], saliva [26], or in a very small sample volume of capillary blood [21].

Since these applications are aimed to be performed in the absence of trained personnel, it is crucial to test our methodology because it provides both the possibility of increased sensitivity and the elimination of the sensors that do not form a proper response.

**Figure 4:**
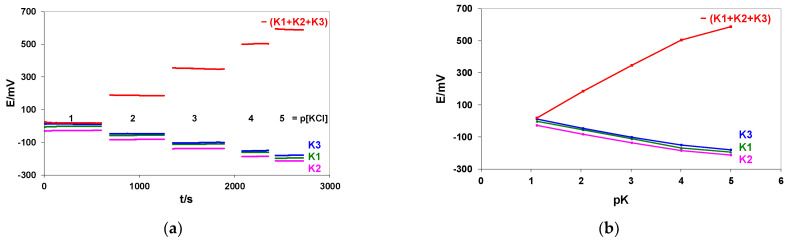
Potential changes of three potassium-sensitive electrodes (K1, K2, and K3) and the sums of their signals recorded during calibration in 0.1–0.00001 M KCl solutions (**a**). Calibration curves for three potassium-sensitive electrodes present in the multielectrode platform and the summed signals (**b**). The multielectrode platform shown in Figure 1b was used.

Our measurements were performed for a flat multiplatform (Figure 1b), containing potassium sensors in 0.1–0.00001 M KCl solutions. The signals of three K-ISs (K1, K2, and K3) were measured versus the SC REF in 30 µL samples and summed. The recorded signals and resulting calibration curves are shown in Figure 4a,b. The independently measured potential difference between the external silver chloride electrode (REF-201, Metrohm) and the SC REF proved the proper functioning of the SC REF (see the last column in Table 3).

The last electrode (K4) from the multielectrode platform was connected simultaneously to another three ion-meter channels with different gains equal to 1.0, 2.0, and 3.2 (to show the additional possibility of slope increasing the EES method, see Appendix A.2).

The detailed calibration results, the calculated correlation coefficients (R), and mean S/N values are summarized in Table 3. The slope values were determined for the linear part of the calibration curves. The potential difference measured between the external silver chloride electrode (REF-201, Metrohm) and the SC REF from the platform demonstrates the proper functioning of the SC REF (see the last column in Table 3).

The results presented in Table 3 have proved again that the proposed method of slope increasing through summing the potentials of three individual K-ISEs brings a 3-times bigger slope value (compared with the signals recorded for single electrodes before summing). Small differences (about 1.8 mV, similar for each KCl concentration) observed between the absolute values of the electronically summed signal and manually summed signals of the K1, K2, and K3 electrodes can be caused by a small offset voltage of the summed amplifier. If one of the electrodes is dysfunctional and discarded from summing, the remaining two will provide the benefit of the EES; in such a case, a 2-times bigger slope.

**Table 3:** Mean potential values (E) with their standard deviation (SD), slope values, calculated correlation coefficients (R), and mean signal-to-noise ratios (S/N) are presented. The last column shows the potential difference between the external silver chloride electrode (REF-201, Metrohm) and SC REF from the platform. The multielectrode platform shown in Figure 1b was used.

pK	E/mV ±SD				
	K1	K2	K3	−(K1 + K2 + K3)	REF Metrohm vs. SC-REF
1.12	−2.43 ± 0.04	−27.19 ± 0.09	10.35 ± 0.09	17.84 ± 0.15	3.74 ± 0.08
2.05	−56.56 ± 0.08	−82.45 ± 0.07	−47.08 ± 0.08	184.39 ± 0.15	3.72 ± 0.09
3.02	−110.94 ± 0.10	−137.56 ± 0.10	−101.03 ± 0.51	347.81 ± 0.51	3.67 ± 0.08
4.01	−168.48 ± 0.30	−186.57 ± 0.07	−150.58 ± 0.65	503.80 ± 0.63	3.34 ± 0.07
5.00	−195.80 ± 0.14	−214.04 ± 0.08	−180.02 ± 0.06	587.80 ± 0.21	3.02 ± 0.06
**Slope mV/pK** **pK 1–4**	−57.45	−55.41	−55.76	168.49	
**R pK 1–4**	−0.99997	−0.99910	−0.99884	0.99955	
**Mean S/N pK 1–4**	730	688	378	711	
**Potential changes between pK 4–5**	−27.32	−27.47	−29.44	84.00	

### 3.2. Extended Benefits: Summing Signals of the Same Electrode Connected Simultaneously to Different Ion-Meter Channels

**Figure 5:**
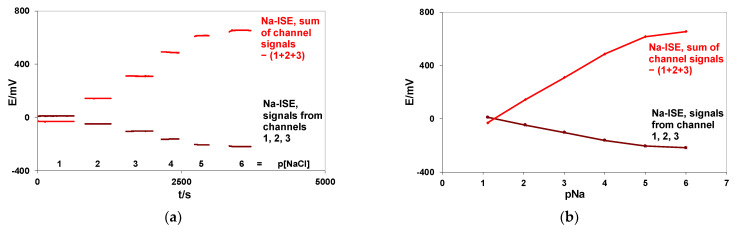
Potential changes of the flow-through sodium electrode connected simultaneously to three different ion-meter channels and their summed signals recorded during calibration in 0.1–0.000001 M NaCl solutions (**a**). Calibration curves of the flow-through sodium electrodes connected simultaneously to three different ion-meter channels and their summed signals (**b**).

It is well known that summing and averaging of subsequently measured signal samples is a good way to minimize random noise [27,28]. It can be expected that the same effect should be observed if the signals of the same electrode, connected simultaneously to different ion-meter channels, are summed up. To check this rule, the signal of a Na-ISE with a plastic membrane was collected simultaneously by three different ion-meter channels, and the resulting output signals of these channels were summed up by a summing amplifier. The calibration was performed in 0.1–0.000001 M NaCl solutions. The potential changes were measured versus the silver chloride electrode REF 201 (Metrohm). The recorded signals and resulting calibration curves are shown in Figure 5a,b.

The same Na-ISE electrode was additionally connected to three another ion-meter channels with different gains equal to 1.0, 2.0, and 3.2 (to show the additional possibility of slope increasing by the EES, see Appendix A.2).

Detailed calibration results, the calculated correlation coefficients (R), and the mean S/N values are presented in Table 4. The slope values were evaluated for linear parts of the calibration curves.

The results presented in Table 4 show that the version of the EES method with summing potentials of the same ISE connected simultaneously to three different ion-meter inputs yield about a 3-times bigger signal value, 3-times bigger slope value, and a mostly improved S/N ratio (compared with signals observed from individual ion-meter inputs). Small differences (about 1.7 mV, similar for each NaCl concentration) observed between the electronically summed signals and absolute values of the manually summed signals from channels 1, 2, and 3 can be caused by a small offset voltage of the summed amplifier.

To summarize, different methods for increasing the slope are obtained either through summing the signals of the “n” electrodes connected to “n” different ion-meter channels (Table 1, Table 2 and Table 3) or the signal of a single electrode connected simultaneously to “n” different ion-meter channels (Table 4) are presented. Experimental results have proved that, as a rule, n-times bigger slope values with mostly improved S/N ratios (in comparison with the single electrode signals measured prior to summation) were observed. Furthermore, as expected, the same effect of the slope increase and S/N ratio improvement were also observed after summing the same electrode signals connected simultaneously to “n” different ion-meter inputs.

It should also be noted that instrumental amplifiers INA116 applied in the input stages of our multichannel ion-meter allow for precise electronic amplification of the electrode signal. To illustrate this possibility of increasing the slope, the potential changes in the chloride electrode connected simultaneously to three ion-meter channels with different gains (1.0, 2.0, and 3.2) are shown in Appendix A.2.

**Table 4:** Mean potential values (E) with their standard deviations (SD), slope values evaluated for the linear parts of the calibration curves, calculated correlation coefficients (R), and signal-to-noise (S/N) ratios are presented. The same flow-through sodium electrode was connected simultaneously to three different ion-meter channels, and the resulting output signals were summed up.

pNa	E/mV ± SD			
	Na-ISE Channel 1	Na-ISE Channel 2	Na-ISE Channel 3	Sum of Channel Signals −(1 + 2 +3)
1.12	9.78 ± 0.05	9.75 ± 0.05	9.73 ± 0.07	−30.49 ± 0.06
2.05	−48.02 ± 0.09	−48.18 ± 0.08	−48.13 ± 0.09	142.76 ± 0.12
3.02	−103.74 ± 0.05	−103.90 ± 0.08	−103.80 ± 0.07	309.76 ± 0.16
4.01	−162.56 ± 0.20	−162.80 ± 0.21	−162.63 ± 0.21	486.03 ± 0.36
5.00	−205.51 ± 0.06	−205.83 ± 0.06	−205.63 ± 0.08	615.09 ± 0.18
6.00	−218.59 ± 0.20	−218.92 ± 0.21	−218.68 ± 0.21	654.26 ± 0.65
**Slope mV/pNa** **pNa 1–4**	−59.54	−59.60	−59.54	178.44
**R pNa 1–4**	−0.99987	−0.99986	−0.99986	0.99987
**Mean S/N pNa 1–4**	835	741	661	1518
**Potential changes** **between pNa 4–5**	−42.95	−43.03	−43.00	129.06
**Potential changes** **between pNa 5–6**	−13.08	−13.09	−13.05	39.17

### 3.3. Comparison of the EES with CCS and SCP Methods

The EES amplification’s benefits are formally identical to those of the CCS method discussed earlier [17,18,19,20]. However, the EES realization is more versatile and prospective, as shown above. The EES advantages originate from the fact that instead of one-electrode readouts in a single cell (the SCP method) or potential read from “n” identical cells (i.e., n ion-sensors and n-reference electrodes) connected in series by the electrochemical bridges (the CCS method), we take the signal from “n” identical electrodes measured vs. one reference electrode. As in the CCS method, there is no need to apply n reference electrodes and n-1 intercell liquid junctions and their contribution to the final measurement error via n-1 liquid junction potentials. The summation of the sensors’ ohmic resistance and its unfavorable contribution to overall resistance does not apply in the EES. Moreover, in the EES, the malfunctioning sensors are discarded before summation, while the signals of the properly functioning ion-sensors are summed, their potentials, including differing formal potentials, averaged, and the total memorized as the calibration data. The system is ready to measure the unknown ionic activity of the analyte in the sample. Compared with a conventional single electrode measurement (SCP), the EES method offers two sides of the same coin regarding advantages: a decreased determination error (DE) for a given potential readout resolution (∆E) and increasing ∆E for the same DE.

## 4. Conclusions

This paper describes a novel and versatile method for increasing the potentiometric sensitivity of ion sensors by modifying the electrical circuits of the measuring device. The proposed method of sensitivity amplification is based on electronic summations of signals taken from the electrodes selected as normally functioning electrodes only. This method lowers the determination error and sample volume.

Our approach demonstrates the benefit of investigating access to a hidden partner in potential measurements, namely, the electric potential amplifying system. We contend that the electrically enhanced sensitivity (EES) strategy complements conventionally performed electrochemical routines known in sensor technology. Furthermore, the EEC electronic system can be supplemented with signal transmitter circuits that allow for remote measurements. Continuous monitoring and/or miniaturization, for example, to serve the background substrate of downscaled, solid-contact, multi-electrode platforms with different architectures, e.g., flat or elastic surfaces, are also highlighted.

It is relevant to expect that the emerging applications of electrochemical ion sensing, namely, bed-side and physiological measurements of electrolytes, flat patch-clamp and cells membrane potential in biophysics, electrochemical tongues in beverages, remote and continuous water control, and last, but not least, industrial processes monitoring, will be the areas of EES practical application. We conclude that narrowing the gap between electronic know-how and electroanalytical applications is the road to success in ion-sensor technology in the non-distant future.

## Figures and Tables

**Figure A2:**
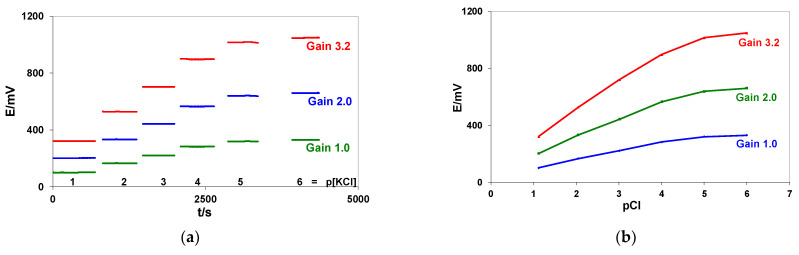
Potential changes of the chloride electrode connected simultaneously to three ion-meter channels with different gains equal to 1.0, 2.0, and 3.2, recorded during calibration in 0.1–0.000001 M KCl solutions (**a**). Calibration curves for chloride electrode connected simultaneously to three ion-meter channels with different gains equal to 1.0, 2.0, and 3.2 (**b**). Potential changes were measured versus the silver chloride electrode REF 251 (Metrohm).

## Appendix A

### Appendix A.1. Summing Amplifier

The inverting summing amplifier was built using an Ultralow Offset Voltage Operational Amplifier OP08A (Analog Device). Three voltage signals can be summed by the proposed circuit, but the number of signals to be summed can be extended by applying additional input resistors R_in_ = 10 kΩ. Summing amplifiers were adapted to function with a custom-built 16-channel ion-meter. An Ultra-Low Input Bias Current Instrumental Amplifier INA116 (Burr Brown) with typical bias current of <3 fA and input resistance of >10^15^ Ω were employed to cooperate with the REF and ISE electrodes.

The scheme in Figure A1 shows the cooperation mode between the multichannel ion-meter, summing amplifier, and 16-bit-resolution data acquisition card (DAC).

A data acquisition card (PCI DAS 6014, Computer Boards; US or USB1606G MC Measurements Computing US) with custom-made software was used for visualization and acquisition of all the measuring signals. The applied software acquires one hundred signal samples from each input (1–4), which were averaged and archived. The procedure was repeated at regular time intervals, programmed by the user. The signals to be summed were delivered to the summing amplifier inputs through R_in1_, R_in2_, and R_in3_ resistors as well as to the measuring card inputs (to inputs 1, 2, and 3 in Figure A1). Simultaneously, the inverted summed signal was delivered to input 4 of the measuring card.

This way, all signals were visualized at the same time, and an improper-functioning electrode(s) to be summed can immediately be detected and ignored in the electrical summing.

In contrast to previously described methods, our proposition allows for summing ISE signals measured versus the same REF electrode. All the electrodes are immersed in the same solution and its volume can be considerably reduced and measurements can be performed even in an extremely small solution volume, e.g., a single drop.

### Appendix A.2. Application of Different Gains for Cl-Ion Sensors

The instrumental amplifiers INA116 applied in the input stages of our multichannel ion-meter allow for precise non-inverting electronic amplification of the electrode signal. The amplification ratio can easily be adjusted by using a single resistor R_G,_ connected between inputs 1 and 16 of the INA116 type. Gain (G) is then described as

G = 1 + 50 kΩ/R_G_

Without R_G_ (i.e., R_G_ = ∞) the gain is equal to 1. A bigger than 1 gain value can be adjusted by the proper choice of R_G_ values. For example, for R_G_ = 74.63 kΩ, the gain is equal to 1.67 and the output voltage changes equal to 100 mV/dec should be observed for electrodes with the Nernstian slope of 59.8 mV. For R_G_ = 21.36 kΩ, similar potential changes will be observed for a divalent electrode with the slope value of 29.9 mV.

To illustrate this possibility of slope increasing, the potential changes in the chloride electrode connected simultaneously to three ion-meter channels with different gains (1.0, 2.0, and 3.2) were recorded versus the silver chloride electrode REF 251 (Metrohm). Calibration was performed in 0.1–0.00001 M KCl solutions.

The recorded potential changes and resulting calibration curves are shown in Figure A2a,b.

Detailed calibration results, the calculated correlation coefficients (R), and mean S/N values are collected in Table A1. The slope values were determined for linear parts of the calibration curves.

Similar results of the Li-ISE, K-ISE, and Na-ISE slope increasing by electronic amplification of the electrode signals were recorded (not shown here)

**Table A1:** Mean potential values (E) with their standard deviations (SD), slope values evaluated for the linear parts of the calibration curves, the calculated correlation coefficients (R), and the signal-to-noise (S/N) ratios are presented. The same chloride electrode was connected simultaneously to three ion-meter channels with different gains equal to 1.0, 2.0, and 3.2.

pCl	E/mV ± SD		
	Cl4 Gain 1.0	Cl4 Gain 2.0	Cl4 Gain 3.2
1.12	100.31 ± 0.08	201.89 ± 0.08	321.20 ± 0.08
2.05	165.27 ± 0.05	331.84 ± 0.09	527.45 ± 0.15
3.02	220.33 ± 0.03	441.96 ± 0.08	720.27 ± 0.09
4.01	281.95 ± 0.13	565.28 ± 0.25	897.98 ± 0.39
5.00	318.94 ± 0.49	639.10 ± 1.01	1015.20 ± 1.62
6.00	329.46 ± 0.15	660.32 ± 0.25	1048.93 ± 0.41
**Slope mV/pCl** **pCl 1–4**	62.35	124.73	199.80
**R pCl 1–4**	0.99917	0.99917	0.99882
**Mean S/N pCl 1–4**	1146	1250	1640
**Potential changes between pCl 4–5**	36.99	73.82	117.22
**Potential changes between pCl 5–6**	10.52	21.22	33.73

As can be seen, amplified signals with an improved S/N ratio and bigger slope values (in comparison with the signals measured with the gain G = 1) were recorded.
